# Genome sequence of *Megasphaera sueciensis* DSM 17042^T^, an obligate anaerobic beer spoiler

**DOI:** 10.1128/mra.00708-25

**Published:** 2025-09-26

**Authors:** Manuel J. Arnold, Matthias A. Ehrmann, Yohanes N. Kurniawan, Koji Suzuki, Wolfgang Liebl

**Affiliations:** 1 Chair of Microbiology, Technical University of Munich 542644, Freising, Germany; 2 Asahi Quality and Innovations Ltd., Moriya, Ibaraki, Japan; Wellesley College, Wellesley, Massachusetts, USA

**Keywords:** beer spoilage, *Megasphaera sueciensis*, genome, obligate anaerobe

## Abstract

*Megasphaera sueciensis* is an obligate anaerobic, gram-negative beer spoilage bacterium causing unwanted off-flavors and turbidity. Here we report the complete and circular genome of 2.9 Mbp with 2,696 predicted coding sequences of the type strain *Megasphaera sueciensis* DSM 17042.

## ANNOUNCEMENT

Beer spoilage caused by *Megasphaera* sp. is a severe and tenacious problem for breweries (1, 2). They produce unwanted turbidity and unpleasant flavors caused by organic acids and short fatty acids (3, 4). *M. sueciensis* is the most recently identified beer spoiler of the genus and has remained the sole representative of its species (4). Even though it was initially isolated from light lager beer in Sweden with an alcohol content of 2.8% (v/v) and a pH of 4.9, the species manages to grow in degassed beers containing up to 7.0% (v/v) ethanol with a pH value of 4.0.

The strain was obtained from the DSMZ (German Collection of Microorganisms and Cell Cultures). It was cultivated at 30°C under anaerobic conditions for 5 days using modified PYG medium (DSM 104). DNA was harvested using the E.Z.N.A. DNA/RNA Isolation Kit (Omega Bio-tek Inc., Norcross, GA, USA) according to the instruction manual with the modification of incubating the sample in the elution buffer heated to 65°C for 5 min prior to elution.

Sequencing was done using Illumina NovaSeq 6000, and the library was prepared using the Illumina DNA Prep Kit (Illumina, San Diego, CA, USA) to generate short reads (S4 PE150 XP; paired-end 2 × 150 bp; Eurofins Genomics Europe Sequencing GmbH, Konstanz, Germany) with 3,881,120 total reads and 1,164,336,000 total bases. SMRT (Single Molecule Real Time) sequencing technology (PacBio RS II, Functional Genomics Center Zurich, ETH Zurich, Switzerland) was used to obtain long reads. The library was created with an insert size of 8–12 kb, delivering at least 200 Mb of raw data from two SMRT cells (1 × 120 min movies) using P4-C2 chemistry. The assembly was executed using SMRT Analysis (v 2.2.0 .p2) and the hierarchical genome assembly process (5). The hybrid assembly was executed via Unicycler (v 0.5.0) (6) in default settings using short and long read data, and the genome sequences were annotated using the NCBI non-WGS Annotation Pipeline PGAP (v 6.4) (7–9). Total sequence length is 2,904,220 bp with a GC content of 40.5% and 2,792 genes (coding ratio of 96.6%), of which 2,696 code for proteins. Furthermore, 4 rRNA operons, 53 tRNAs, 4 non-coding RNA operons, and 2 CRISPR arrays were found. The strain possesses putative genes coding for amino acid decarboxylases (arginine, ornithine) possibly functioning as an acid stress response. The genome harbors several genes annotated as efflux transporters or alcohol dehydrogenases that might be involved in stress response or in degradation of sugar alcohols. Putative genes encoding proteins resembling known hop transporters HorA, HitA, and Nramp were found that are described to convey hop tolerance (10, 11).

## Data Availability

The assembled genome for DSM 17042^T^ (TMW 2.2488) is available under accession number CP116939.1, with BioProject accession number PRJNA868947. The raw reads are available under SRA accession numbers SRX28880809 and SRX29435982.
